# LncRNA NEAT1/microRNA-129-5p/SOCS2 axis regulates liver fibrosis in alcoholic steatohepatitis

**DOI:** 10.1186/s12967-020-02577-5

**Published:** 2020-11-24

**Authors:** Junfeng Ye, Yuanqiang Lin, Ying Yu, Di Sun

**Affiliations:** 1 Department of Hepato-Biliary-Pancreatic Surgery, First Hospital, Jilin University, Changchun, 130021 Jilin People’s Republic of China; 2 Department of Ultrasonography, China-Japan Union Hospital, Jilin University, Changchun , 130021 Jilin People’s Republic of China; 3 Department of Colorectal & Anal Surgery, First Hospital, Jilin University, No. 71 Xinmin street, Changchun, 130021 Jilin People’s Republic of China

**Keywords:** Alcoholic steatohepatitis, Long non-coding RNA nuclear paraspeckle assembly transcript 1, MicroRNA-129-5p, Suppressor of cytokine signaling 2, Liver fibrosis

## Abstract

**Background:**

Long non-coding RNA nuclear paraspeckle assembly transcript 1 (NEAT1) has been reported to play an essential role in non-alcoholic fatty liver disease. However, the role of NEAT1 in regulation of alcoholic steatohepatitis (ASH) remains largely unknown. This study aims to explore the role of NEAT1 in ASH by mediating microRNA-129-5p (miR-129-5p) targeting suppressor of cytokine signaling 2 (SOCS2).

**Methods:**

NEAT1, miR-129-5p and SOCS2 expression in serum of ASH patients were assessed. In the in vitro cellular experiment, we transfected siRNAs, oligonucleotides or plasmids into ethanol-induced AML-12 mouse hepatocytes to alter NEAT1 and miR-129-5p expression, and inflammatory factors and lipid content were determined. In the in vivo animal experiment, we injected lentiviruses carrying siRNAs, oligonucleotides or plasmids onto ASH mice (ASH induced by feeding mice a Lieber-DeCarli ethanol diet) to alter NEAT1 and miR-129-5p expression through the tail vein. Serum liver function, blood lipids and inflammatory factors were detected; liver histopathology, liver cell apoptosis, and fibrosis were observed. The relationship between NEAT1 and miR-129-5p, or between miR-129-5p and SOCS2 was verified.

**Results:**

MiR-129-5p was reduced while NEAT1 and SOCS2 were elevated in ASH. Inhibited NEAT1 or elevated miR-129-5p suppressed the elevated lipid metabolism and restrained inflammation reaction in ethanol-stimulated AML-12 cells. The promoted miR-129-5p and inhibited NEAT1 could improve the liver function and repress blood lipid, inflammation reaction, hepatocyte apoptosis and liver fibrosis in ethanol-induced ASH mice. Furthermore, NEAT1 could negatively regulate miR-129-5p to target SOCS2.

**Conclusion:**

We have found that the inhibited NEAT1 could suppress liver fibrosis in ASH mice by promoting miR-129-5p and restraining SOCS2, thereby decelerating the development of ASH.

## Background

As the largest solid organ, liver serves as a pivotal role in metabolism and detoxication in human body. Liver injury is the main cause that greatly contributes to the risk of human health globally, especially in Asian countries [1]. Second to viral hepatitis, alcoholic liver disease (ALD) and non-alcoholic fatty liver disease (NAFLD) are the commonest liver diseases in developed countries [2]. ALD is a deleterious result of excessive alcohol consumption, resulting in a great burden and a loss of socioeconomic productivity in the world [3]. Alcohol consumption would promote lipogenesis and mobilization of lipids and decelerate lipid catabolism, causing a lipid accumulation in hepatocytes, called fatty liver. In some cases, alcohol consumption results in an inflammatory reaction, which is known as alcoholic steatohepatitis (ASH) if there is hepatic lipid deposition [4]. Although ASH progresses slowly, the continual chronic liver injury and inflammation would finally lead to progressive fibrosis and cirrhosis, then may promote the progression of hepatocellular carcinoma (HCC) [4]. As an acute inflammatory liver disease associated with poor prognosis [5], novel and efficient treatment for ASH is urgently needed.

Long noncoding RNAs (lncRNAs) are a kind of non-coding RNAs of over 200 nucleotides and have little or no protein-coding ability [6]. Abnormal expression of particular lncRNAs has been demonstrated to be related with development of human diseases. LncRNA nuclear paraspeckle assembly transcript 1 (NEAT1) is a novel lncRNA that transcribed from many endocrine neoplasia locus [7]. As reported, NEAT1 promoted inflammatory response in sepsis-induced liver injury [8], and NEAT1 also facilitated hepatic lipid accumulation and exacerbated NAFLD [9, 10]. Furthermore, microRNAs (miRNAs) regulate expression of target gene through binding to the 3′-untranslated region (3′UTR) of target gene mRNA, causing degradation or translational suppression of mRNA [11]. Multiple miRNAs have been identified in ASH, including miR-31, miR-191 and miR-451 [12]. As a member of miRNAs, miR-129-5p has been identified to regulate the progression of HCC [13, 14]. In addition, the regulatory relation between NEAT1 and miR-129-5p has been revealed in HCC [15] and hepatoblastoma [16], while this relation has not been unraveled in ASH. Additionally, suppressor of cytokine signaling (SOCS) family was initially recognized as suppressors of the cytokine signaling [17]. As one of the SOCS family, SOCS2 was clarified as a feedback suppressor of the growth hormone/insulin-like growth factor axis [18]. It has been clarified that SOCS2 participated in NAFLD [19] and hepatic steatosis [20]. Nevertheless, the impact of SOCS2 on ASH, as well as the target relation between SOCS2 and miR-129-5p remains to be further explored. This study was designed to elucidate the role of the NEAT1/miR-129-5p/SOCS2 axis in the progression of ASH, and we inferred that the inhibited NEAT1 may repress liver fibrosis in ASH mice by regulating miR-129-5p and SOCS2.

## Materials and methods

### Ethics statement

Written informed consents were acquired from all patients before this study. The protocol of this study was confirmed by the Ethic Committee of First hospital of Jilin university. The protocol of animal experiments was approved by the Institutional Animal Care and Use Committee of First hospital of Jilin university.

### Study subjects

Sixty-five cases of ASH patients (47 males and 18 females, aging 21–66 years, mean age of 39.82 ± 6.07 years) who were in line with the diagnostic criteria of ASH [21] and accepted treatment in First hospital of Jilin university from April 2017 to March 2018 were selected. Patients with diabetes, coronary heart disease, hyperlipidemia, hypertension, or other cardio-cerebral vascular diseases; malignant tumor, viral hepatitis, drug-induced hepatitis, or other non-alcoholic hepatic injury; women during pregnant stage or breast-feed stage, or patients with a poor compliance were excluded. Sixty-five people with healthy examination (41 males and 24 females, mean age of (41.75 ± 8.26 years) were also collected. Peripheral venous blood (5 mL) from ASH patients and people with healthy examination were preserved at − 4 °C for 30 min and centrifuged to collect serum, in which alanine aminotransferase (ALT), aspartate aminotransferase (AST), total cholesterol (TC) and triglyceride (TG) in serum were determined by an automatic biochemical analyzer and its original kit (Shenzhen Mindray Software Technology Co., Ltd., Shenzhen, China), as well as tumor necrosis factor-α (TNF-α), interleukin-1β (IL-1β) and IL-6 in serum by enzyme-linked immunosorbent assay (ELISA) kits (Beckman Coulter Life Sciences, Brea, CA, USA).

### Cell culture and treatment

Mouse liver cell line AML-12 was purchased from Shanghai Institute of Biochemistry and Cell Biology, Chinese Academy of Sciences (Shanghai, China) and cultured in DMEM-F12 medium containing 10% fetal bovine serum (FBS). Upon cell adherence, they were passaged the second day, and those in the logarithmic growth phase were used in the experiments.

AML-12 cells were stimulated by 100 mM ethanol for 24 h alone, or stimulated by 100 mM ethanol for 24 h, followed by transfection of siRNA-NEAT1 negative control (NC), siRNA-NEAT1, miR-129-5p mimic NC, miR-129-5p mimic, NEAT1 overexpression vector (overexpressed (oe)-NEAT1) together with miR-129-5p mimic NC, or oe-NEAT1 together with miR-129-5p mimic. The siRNA-NC, siRNA-NEAT1, mimic-NC, miR-129-5p mimic and oe-NEAT1 were all acquired from GenePharma Co., Ltd, (Shanghai, China).

AML12 cells were seeded in 12-well plates and cultured for 24 h before transfection. The transfection was conducted by Lipofectamine 2000 reagent (Invitrogen Inc., Carlsbad, CA, USA) when the cell confluence reached 70–90%. Transfected for 6 h, the medium was changed and cells were stimulated by 100 mM ethanol for 24 h. Finally, cells and cell supernatant were collected for the subsequent cell experiments.

### 3-(4,5-dimethyl-2-thiazolyl)-2,5-diphenyl-2-H-tetrazolium bromide (MTT) assay

Cells were detached and made into single cell suspension, which was seeded onto 96-well plates at 100 μL/well (1.0 × 10^4^ cells/well), and the marginal wells were appended with phosphate buffered saline (PBS). The adherent cells were stimulated by different concentrations of ethanol (6 duplicates were set) and cultured for 24 h. Then, cells were supplemented with 20 μL MTT solution (5 mg/mL), wrapped by silver paper and then cultured for 4 h. After that, cells were added with 150 μL dimethyl sulfoxide and cultured without light exposure for 10 min. The optical density value was analyzed by a microplate reader.

### Experimental animals

A total of 80 male C57BL/6 mice (aging 4 w, weighing 18–20 g) that acquired from Laboratory Animal Center of Jilin University (Changchun, China) were adaptively fed at 20–25℃ and 55% humidity in a clean environment for 1 w, with free access to food and water and normal 12 h day/night cycle.

### Establishment of ASH mice models

The mice were fed with Lieber-DeCarli ad libitum diet (Dyets Inc. Bethlehem, PA, USA) and 5% (vol/vol) ethanol (36% ethanol-derived calories) for 4–7 w, and were given 20% ethanol (5 g/kg, body weight) by gavage. Mice for control (n = 10) were fed and given maltodextrin (same calories with the ethanol). The fodder was freshly configured every day and gavage was conducted in the last day of the modeling [22].

### Animal treatment

The 70 successfully modeled mice were injected with lentiviruses expressing siRNA-NC, siRNA-NEAT1, agomiR-NC, miR-129-5p agomiR, Oe-NEAT1 + agomiR-NC, or Oe-NEAT1 + miR-129-5p agomiR (each 100 μL) [23] at caudal vein. The aforementioned lentivirus vector or agomir was constructed by GenePharma. After 4-w continuous injection, mice were euthanized to collect eyeball blood and liver tissues.

### Determination of serum factors

The supernatant of AML-12 cells and mouse eyeball blood were collected, in which ALT and AST were determined by a fully automatic biochemical analyzer and its matching kits (both from Mindray Biomedical Electronics Co., Ltd., Guangdong, China), TC and TG by microplate reader colorimetry (microplate reader was purchased from Shanghai Labsystems Biotechnology Co., Ltd., Shanghai, China) and TNF-α, IL-1β and IL-6 contents by ELISA kits (Beckman Coulter Life Sciences).

### Hematoxylin–eosin (HE) staining and oil red O staining

HE staining: the mouse liver tissues were fixed, embedded, sectioned and stained. Liver tissues (1 cm × 1 cm) were fixed with 4% paraformaldehyde for 24 h, dehydrated by gradient ethanol, permeabilized by 1/2 xylene and 1/2 ethanol, embedded by paraffin and sectioned. The sections were stained by hematoxylin for 15 min, differentiated by hydrochloric alcohol for 10 s, washed by tap water for 1 min, stained by 0.5% eosin for 3 min, and then soaked in ethanol for 5 min. After permeabilized and sealed, the sections were observed under a microscope.

Oil red O staining: mouse liver tissues were embedded, sectioned and stained. The pathology of liver tissues was observed under a light exposure. The sections (10-μm) were dried for 15 min, incubated by 100% isopropanol for 5 min, stained by 0.5% oil red O solution for 10 min, toasted at 60℃, and then rinsed by 85% isopropanol solution for 3 min. After counterstained by hematoxylin for 2 min, the sections were sealed by glycerin gelatin and observed under a microscope.

### Terminal deoxynucleotidyl transferase-mediated dUTP nick end-labeling (TUNEL) staining

The cell apoptosis in liver tissue was determined by TUNEL staining (Beijing Zhongshu GoldenBridge Biotechnology Co., Ltd., Beijing, China). The sections were developed by diaminobenzidine and counterstained by hematoxylin, then the apoptotic cells were observed under a light microscope (Olympus Optical Co., Ltd, Tokyo, Japan). Apoptotic index (AI) = the number of apoptotic cells/the number of total cells × 100%.

### Masson’s staining

The tissues were normally sectioned, dewaxed, dehydrated and stained by Masson kits (Service Biological Technology, Wuhan, China). The collagen deposition was observed using a light microscope and the images were analyzed by the Image-Pro Plus software.

### Reverse transcription quantitative polymerase chain reaction (RT-qPCR)

Trizol reagent (Life Technologies, Carlsbad, CA, USA) was employed to extract total RNA in serum, tissues and cells. RNA was reversely transcribed into cDNA via RT-qPCR kits (Takara, Kusatsu, Shiga, Japan). PCR was conducted by SYBR Green PCR Master Mix kits (Roche, Palo Alto, CA, USA) and the data were analyzed by 2^−ΔΔCt^ method [24]. U6 was taken as the loading control of miR-129-5p and glyceraldehyde phosphate dehydrogenase (GAPDH) of NEAT1, SOCS2, collagen-I, and collagen-III. The primers (Table 1) were synthesized by Invitrogen.

**Table 1:** Primer sequence

Gene	Sequence
NEAT1	F: 5′-CTCACTAAAGGCACCGAAG-3′
	R: 5′-GGCAGAGAAGTTGCTTGTGG-3′
MiR-129-5p	F: 5′-CACTTGGCTGCCCGATACTCT-3′
	R: 5′-GCCCATGTTGTCCTGGATGTT-3′
SOCS2	F: 5′-AGCAGTTTGACAGCGTGGTT-3′
	R: 5′-CAGGTAAAGGTGAACAGTCCC-3′
Collagen-I	F: 5′-GAGGGCCAAGACGAAGACATC-3′
	R: 5′-CAGATCACGTCATCGCACAAC-3′
Collagen-III	F: 5′-GGAGCTGGCTACTTCTCGC-3′
	R: 5′-GGGAACATCCTCCTTCAACAG-3′
U6	F: 5′-GCTCGCTTCGGCAGCACA-3′
	R: 5′-GAGGTATTCGCACCAGAGGA-3′
GAPDH	F: 5′-ACGGCAAGTTCAACGGCACAG-3′
	R: 5′-GACGCCAGTAGACTCCACGACA-3′

*F* forward, *R* reverse, *NEAT1* nuclear paraspeckle assembly transcript 1, *miR-129-5p* microRNA-129-5p, *SOCS2* suppressor of cytokine signaling 2, *GAPDH* glyceraldehyde phosphate dehydrogenase

### Western blot analysis

Total proteins in serum, tissues and cells were evaluated by bicinchoninic acid kits (Pierce, Rockford, IL, USA). The extracted proteins that had been denatured by high temperature were mixed with loading buffer, boiled at 95 °C for 10 min, conducted with 10% sodium dodecyl sulfate–polyacrylamide gel electrophoresis and transferred onto the polyvinylidene fluoride membranes. After fixed with 5% bovine serum albumin (10-L16, Beijing Zhongshenglikang Technology Co., Ltd., Beijing, China), the membranes were appended with primary antibodies SOCS2 (1: 300, Abcam Inc., Cambridge, MA, USA) and GAPDH (1: 1000, Cell Signaling Technology, Beverly, MA, USA), and the horseradish peroxidase-conjugated secondary antibody (1: 5000; ab6721, Abcam). Next, the membranes were developed by enhanced chemiluminescent reagent and exposed in a dark room. GAPDH was taken as the internal reference and the data were analyzed by the Bio-Rad Image Lab system (GEL DOC EZ IMAGER, Bio-rad, CA, USA). Gray values of the protein bands were analyzed by the Image J software.

### Dual luciferase reporter gene assay

The binding sites of NEAT1 and miR-129-5p, together with miR-129-5p and SOCS2 were predicted and analyzed by bioinformatic websites https://starbase.sysu.edu.cn/ and https://www.targetscan.org/vert_72/. The predicted binding sequences and their corresponding mutated sequence were inserted into pmirGLO vector (Promega, Madison, WI, USA) to build pmirGLO-NEAT1-wild type (Wt), pmirGLO-NEAT1-mutant (Mut), pmirGLO-SOCS2-Wt, and pmirGLO-SOCS2-Mut. Meanwhile, the Wt and Mut vectors were co-transfected with mimic-NC or miR-129-5p mimic into 293 T cells for 24 h. Luciferase activity was detected by Dual-Lucy Assay Kit (Solarbio, Beijing, China).

### RNA-pull down assay

NEAT1-Wt and NEAT1-Mut (50 nM) labeled by biotin were severally transfected into cells for 48 h and incubated by particular cell lysis buffer (Ambion Company, Austin, TX, USA) for 10 min. Next, 50 mL cell lysate was sub-packaged and the rest lysate was co-cultured with M-280 streptavidin beads (Sigma-Aldrich, St Louis, MO, USA) coated with RNase-free and yeast tRNA (Sigma-Aldrich). Total RNA was extracted by Trizol and miR-129-5p level was evaluated by RT-qPCR.

### Statistical analysis

All data analyses were conducted using SPSS 21.0 software (IBM Corp. Armonk, NY, USA). The measurement data were expressed as mean ± standard deviation, and Pearson correlation analysis was employed for correlation test. The unpaired t-test was performed for comparisons between two groups, one-way analysis of variance (ANOVA) for those among multiple groups and Tukey’s post hoc test for pairwise comparisons. *P* value < 0.05 was indicative of statistically significant difference.

## Results

### NEAT1 and SOCS2 are highly expressed while miR-129-5p is poorly expressed in serum of ASH patients

The levels of corresponding indicators of ASH patients and healthy controls were evaluated (Table 2), and the results reflected that body mass index (BMI), ALT, AST, TC, TG, TNF-α, IL-1β and IL-6 levels were higher in ASH patients.

**Table 2:** Levels of corresponding indicators of ASH patients and healthy people

Indicators	The ASH group (n = 65)	The healthy group (n = 65)	*P*
BMI (Kg/m^2^)	28.57 ± 5.26	22.49 ± 4.32	< 0.001
ALT (U/L)	33.57 ± 12.16	19.75 ± 10.21	< 0.001
AST (U/L)	62.79 ± 24.51	26.14 ± 13.87	< 0.001
TC (mmol/L)	5.39 ± 0.94	4.02 ± 0.76	< 0.001
TG (mmol/L)	2.68 ± 0.83	1.47 ± 0.66	< 0.001
TNF-α (pg/mL)	149.75 ± 21.62	25.68 ± 6.03	< 0.001
IL-1β (pg/mL)	54.82 ± 6.77	23.25 ± 2.41	< 0.001
IL-6 (pg/mL)	211.35 ± 49.08	40.90 ± 11.37	< 0.001

*ASH* alcoholic steatohepatitis, *BMI* body mass index, *ALT* alanine aminotransferase, *AST* aspartate aminotransferase, *TC* total cholesterol, *TG* triglyceride, TNF-α tumor necrosis factor-α, *IL-1β* interleukin-1β, *IL-6* interleukin-6

**Fig. 1:**
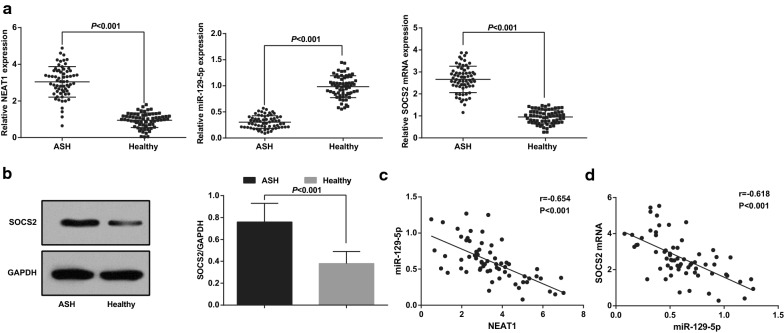
LncRNA NEAT1 and SOCS2 are highly expressed while miR-129-5p is poorly expressed in serum of ASH patients. A, expression of NEAT1, miR-129-5p, and SOCS2 in the ASH group and the healthy group; B, protein expression of SOCS2 in the ASH group and the healthy group; C, relation between the expression of miR-129-5p and NEAT1 in serum of ASH patients was analyzed by Pearson correlation analysis; D, relation between the expression of miR-129-5p and SOCS2 mRNA in serum of ASH patients was analyzed by Pearson correlation analysis; n = 65, the measurement data were expressed as mean ± standard deviation, and the unpaired t-test was performed for comparisons between two groups

NEAT1, miR-129-5p and SOCS2 in serum of ASH patients and healthy controls were determined by RT-qPCR and Western blot analysis. The outcomes indicated that (Fig. 1a, b) NEAT1 and SOCS2 levels were elevated while miR-129-5p expression was inhibited in ASH patients.

The relations between NEAT1 and miR-129-5p, and between miR-129-5p and SOCS2 mRNA in serum of ASH patients were analyzed by Pearson correlation analysis (Fig. 1c, d), and the results mirrored that NEAT1 and miR-129-5p were in a negative relation (r = − 0.654, *P* < 0.001), and miR-129 was also negatively related with SOCS2 mRNA (r = − 0.618, *P* < 0.001).

### Inhibited NEAT1 or elevated miR-129-5p inhibits the elevated lipid metabolism and restrains inflammation reaction in ethanol-stimulated AML-12 cells

**Fig. 2:**
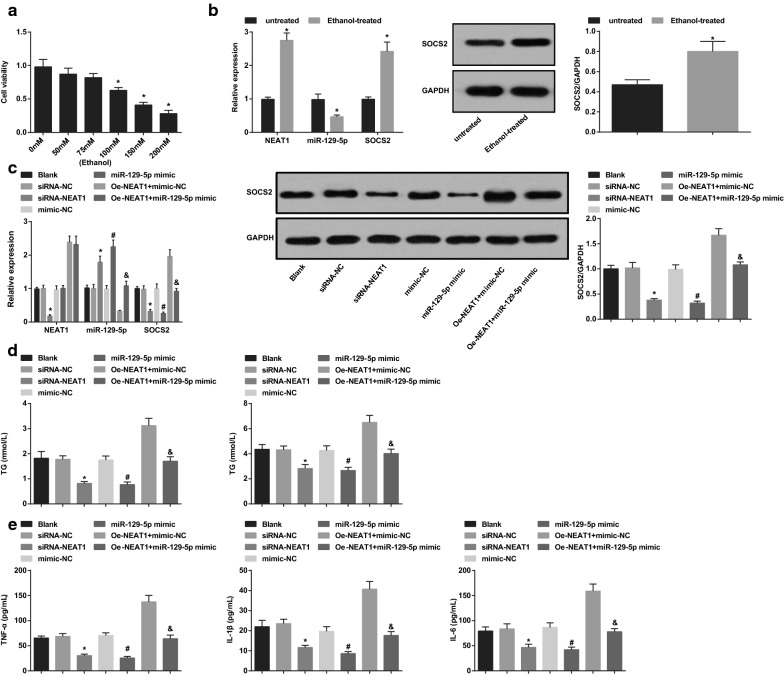
Inhibited NEAT1 or elevated miR-129-5p inhibits the elevated lipid metabolism and restrains inflammation reaction in ethanol-stimulated AML-12 cells. A, impacts of different concentrations of ethanol on AML-12 cell viability were evaluated by MTT assay; B, the expression of NEAT1, miR-129-5p, and SOCS2 in AML-12 cells in the ethanol untreated group and the ethanol treated group; C, the expression of NEAT1, miR-129-5p, and SOCS2 in AML-12 cells that have been treated with ethanol after transfection; D, contents of TC and TG in AML-12 cells that have been treated with ethanol after transfection; E, contents of TNF-α, IL-1β, and IL-6 in AML-12 cells that have been treated with ethanol after transfection; **a** *P < 0.05 vs the AML-12 cells that have not been stimulated by ethanol; **b** *P < 0.05 vs the untreated group; **c**–**e** *P < 0.05 vs the siRNA-NC group, ^#^*P* < 0.05 *vs* the mimic-NC group, ^&^*P* < 0.05 vs the oe-NEAT1 + mimic-NC group; N = 3, the measurement data were expressed as mean ± standard deviation, one-way ANOVA was used for comparisons among multiple groups, and Tukey’s post hoc test was used for pairwise comparisons after one-way ANOVA

MTT assay observed the effect of different concentrations of ethanol on AML-12 cell viability, and the results (Fig. 2a) revealed that ethanol over 100 mM concentration indeed impaired cell viability. Hence, 100 mM ethanol was chosen in the subsequent experiments in vitro. NEAT1, miR-129-5p and SOCS2 expression in ethanol-treated AML-12 cells were determined by RT-qPCR and Western blot analysis and the outcomes (Fig. 2b) suggested that NEAT1 and SOCS2 expression were increased while miR-129-5p expression was reduced.

AML-12 cells were transfected with siRNAs, oligonucleotides or plasmids and stimulated by ethanol for 24 h. It was demonstrated that NEAT1 and SOCS2 expression was decreased while miR-129-5p expression was increased by NEAT1 down-regulation. SOCS2 expression was reduced but miR-129-5p expression was advanced by miR-129-5p up-regulation. Up-regulating miR-129-5p reversed the effect of highly expressed NEAT1 on SOCS2 expression in AML-12 cells (Fig. 2c).

Measurement of lipid content and inflammatory factors mirrored that (Fig. 2d, e) down-regulating NEAT1 or up-regulating miR-129-5p reduced TC, TG, TNF-α, IL-1β and IL-6. Restoring miR-129-5p reversed the effect of overexpressed NEAT1 on lipid content and inflammatory factors in ethanol-stimulated AML-12 cells.

### Inhibited NEAT1 or elevated miR-129-5p promotes liver function and suppresses blood lipid and inflammation reaction in ASH mice

**Fig. 3:**
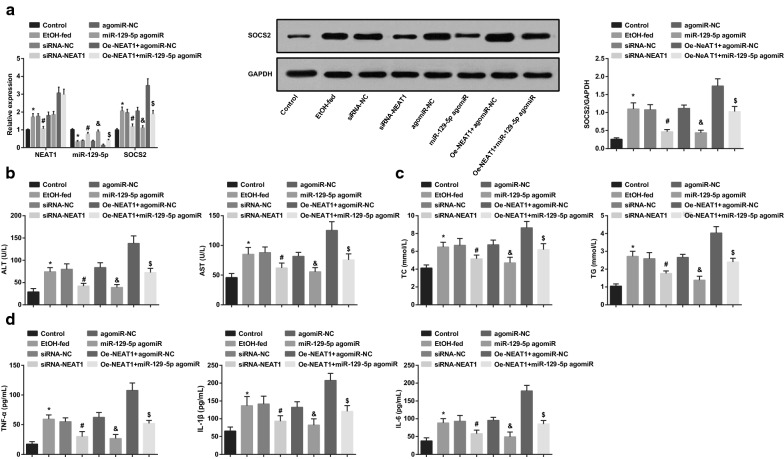
Inhibited NEAT1 or elevated miR-129-5p suppresses blood lipid and inflammation reaction in ASH mice. A, expression of NEAT1, miR-129-5p, and SOCS2 in mice’s liver tissues of each group; B, contents of ALT and AST in mice’s serum in each group; C, contents of TG and TC in mice’s serum in each group; D, contents of TNF-α, IL-1β, and IL-6 in mice’s serum in each group; *P < 0.05 vs the control group, ^#^*P* < 0.05 vs the siRNA-NC group, ^&^*P* < 0.05 vs the agomiR-NC group, ^$^*P* < 0.05 vs the oe-NEAT1 + agomiR-NC group; n = 10, the measurement data were expressed as mean ± standard deviation, one-way ANOVA was used for comparisons among multiple groups, and Tukey’s post hoc test was used for pairwise comparisons after one-way ANOVA

NEAT1, miR-129-5p and SOCS2 expression in liver tissues of ASH mice were measured and the outcomes (Fig. 3a) indicated that NEAT1 and SOCS2 expression were elevated while miR-129-5p expression was repressed in ASH mice. Silencing NEAT1 restricted NEAT1 and SOCS2 expression and promoted miR-129-5p expression. Enhancing miR-129-5p reduced SOCS2 expression but raised miR-129-5p expression. miR-129-5p elevation reversed the effect of overexpressed NEAT1 on SOCS2 expression in ASH mice.

The levels of serum lipid of mice were determined, and we have found that (Fig. 3b–d) TC, TG, TNF-α, IL-1β and IL-6 contents were increased in ASH mice, which were suppressed by knocking down NEAT1 or elevating miR-129-5p. miR-129-5p restoration reversed the effect of NEAT1 overexpression on blood lipid and inflammation reaction. These finding suggested that the silenced NEAT1 or up-regulated miR-129-5p was able to promote the liver function and repress blood lipid and inflammation reaction in ASH mice.

### Inhibited NEAT1 or elevated miR-129-5p relieves pathology of liver tissues and apoptosis of hepatocytes in ASH mice

**Fig. 4:**
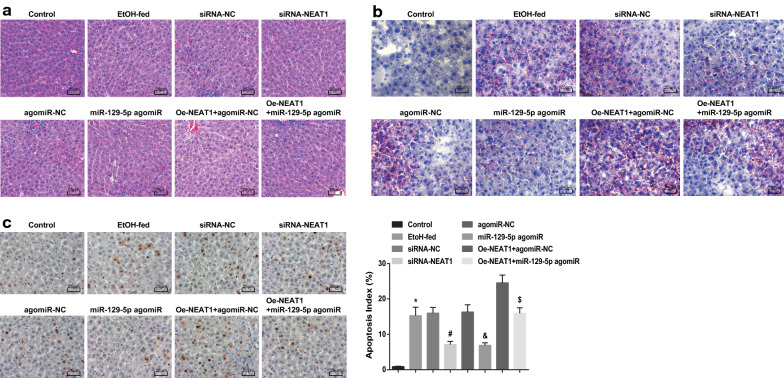
Inhibited NEAT1 or elevated miR-129-5p relieves pathology of liver tissues and apoptosis of hepatocytes in ASH mice. A, representative images of HE staining; B, representative images of oil red O staining; C, representative images of TUNEL staining and the hepatocytes apoptosis of each group of mice; *P < 0.05 vs the control group, ^#^*P* < 0.05 vs the siRNA-NC group, ^&^*P* < 0.05 vs the agomiR-NC group, ^$^*P* < 0.05 vs the oe-NEAT1 + agomiR-NC group; n = 10, the measurement data were expressed as mean ± standard deviation, one-way ANOVA was used for comparisons among multiple groups, and Tukey’s post hoc test was used for pairwise comparisons after one-way ANOVA

The pathology of mouse liver tissue and lipid droplets were observed by HE staining and oil red O staining. The outcomes revealed that (Fig. 4a, b) the hepatocytes in liver tissues of normal mice were normal and orderly arranged, and there was no lipid vacuole or obvious lipid droplet. In ASH mice or AHS mice that were treated with lentiviruses expressing siRNA-NC, agomiR-NC or oe-NEAT1 + miR-129-5p agomiR, there were necrotic tissues, blurred liver lobule structure, abundant lipid vacuoles and inflammatory cell infiltration, as well as increased lipid droplets. In AHS mice that were treated with lentiviruses expressing siRNA-NEAT1 or miR-129-5p agomiR, the steatosis, inflammation reaction and cell necrosis were abated and lipid droplets were decreased. The pathological damage was aggravated and lipid droplets were further augmented in AHS mice that were treated with lentiviruses expressing oe-NEAT1 + agomiR-NC.

The apoptosis of hepatocytes in mouse liver tissue was observed by TUNEL staining. The nuclei of the apoptotic hepatocytes were stained into brown and we have found that (Fig. 4c) ASH mice showed increased AI, which were reduced by down-regulated NEAT1 or up-regulated miR-129-5p. miR-129-5p enhancement attenuated the effect of NEAT1 overexpression on AI of hepatocytes. It was hinted that inhibited NEAT1 or elevated miR-129-5p could relieve pathology of liver tissues and apoptosis of hepatocytes in ASH mice.

### Inhibited NEAT1 or elevated miR-129-5p ameliorates liver fibrosis in ASH mice

**Fig. 5:**
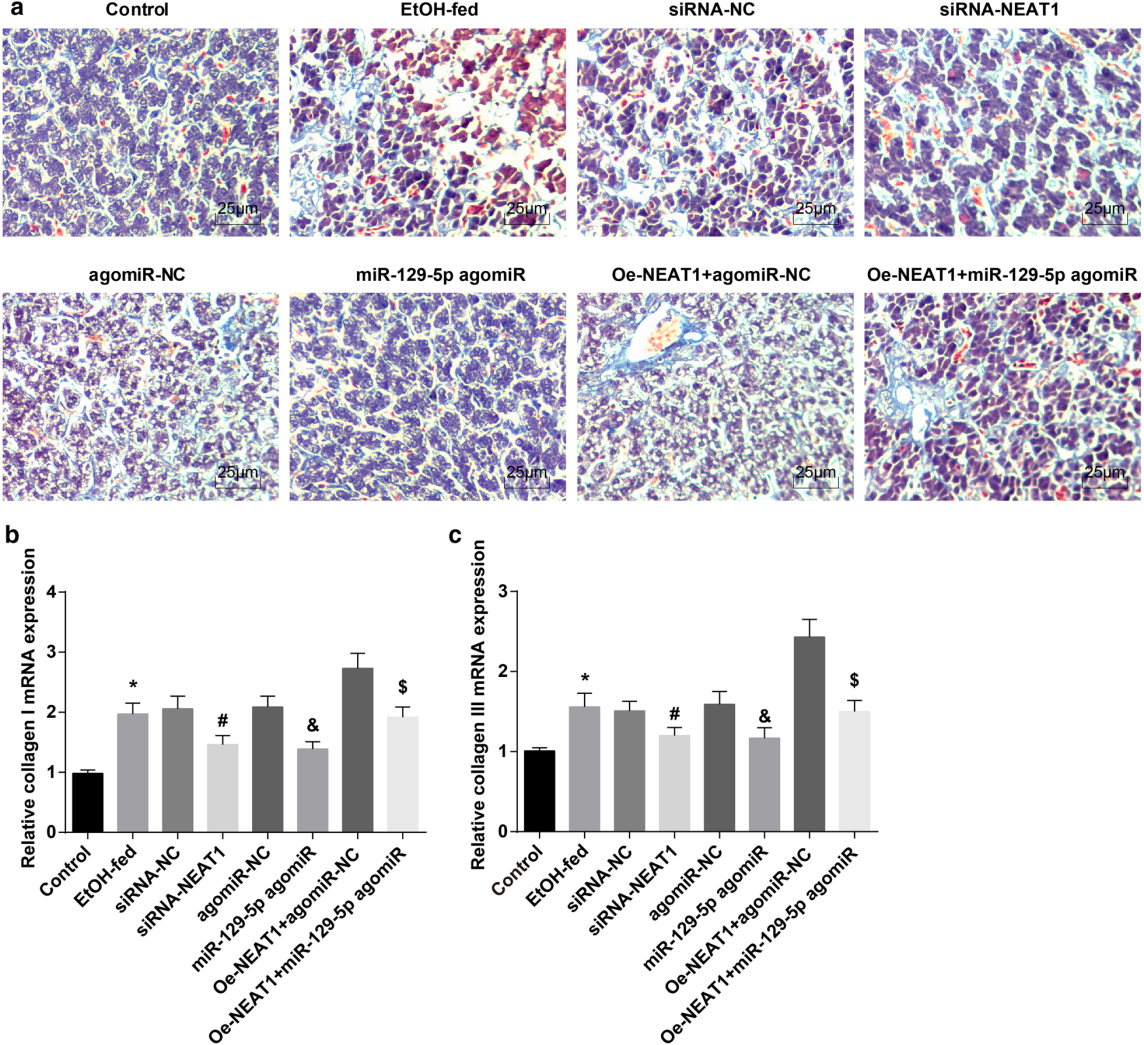
Inhibited NEAT1 or elevated miR-129-5p ameliorates liver fibrosis in ASH mice. **a** representative images of Masson’s staining; **b** expression of collagen-I in mice’s liver tissues in each group; **c** expression of collagen-III in mice’s liver tissues in each group; *P < 0.05 vs the control group, ^#^*P* < 0.05 vs the siRNA-NC group, ^&^*P* < 0.05 vs the agomiR-NC group, ^$^*P* < 0.05 vs the oe-NEAT1 + agomiR-NC group; n = 10, the measurement data were expressed as mean ± standard deviation, one-way ANOVA was used for comparisons among multiple groups, and Tukey’s post hoc test was used for pairwise comparisons after one-way ANOVA

Masson’s staining was employed to observe the fibrous hyperplasia of mouse liver tissues and the outcomes (Fig. 5a) suggested that there were plump and orderly arranged hepatocytes, clear liver lobule structure, infinitesimal blue in central vein and blood vessel wall of portal area, and no obvious collagen fiber hyperplasia in liver tissues of the normal mice. In ASH mice or AHS mice that were treated with lentiviruses expressing siRNA-NC, agomiR-NC or oe-NEAT1 + miR-129-5p agomiR, there were abundant blue adherence in portal area, increased and broadened fibrous septum connected into bridges, fake lobule formed by fibrous septum, and obvious collagen fiber hyperplasia in mouse liver tissues. The collagen fiber hyperplasia and hyperplasia of connective tissues around the central vein and portal area were reduced in liver tissues of AHS mice that were treated with lentiviruses expressing siRNA-NEAT1 or miR-129-5p agomiR while increased in AHS mice that were treated with lentiviruses expressing oe-NEAT1 + agomiR-NC.

The levels of fibrous factors in mouse liver tissue presented that (Fig. 5b, c) collagen-I and collagen-III expression were enhanced in ASH mice, which would be attenuated by suppressing NEAT1 or increasing miR-129-5p. Up-regulating miR-129-5p reversed the effect of NEAT1 overexpression on fibrosis-related factors in liver tissues of ASH mice.

### MiR-129-5p particularly binds with NEAT1, and SOCS2 is targeted by miR-129-5p

**Fig. 6:**
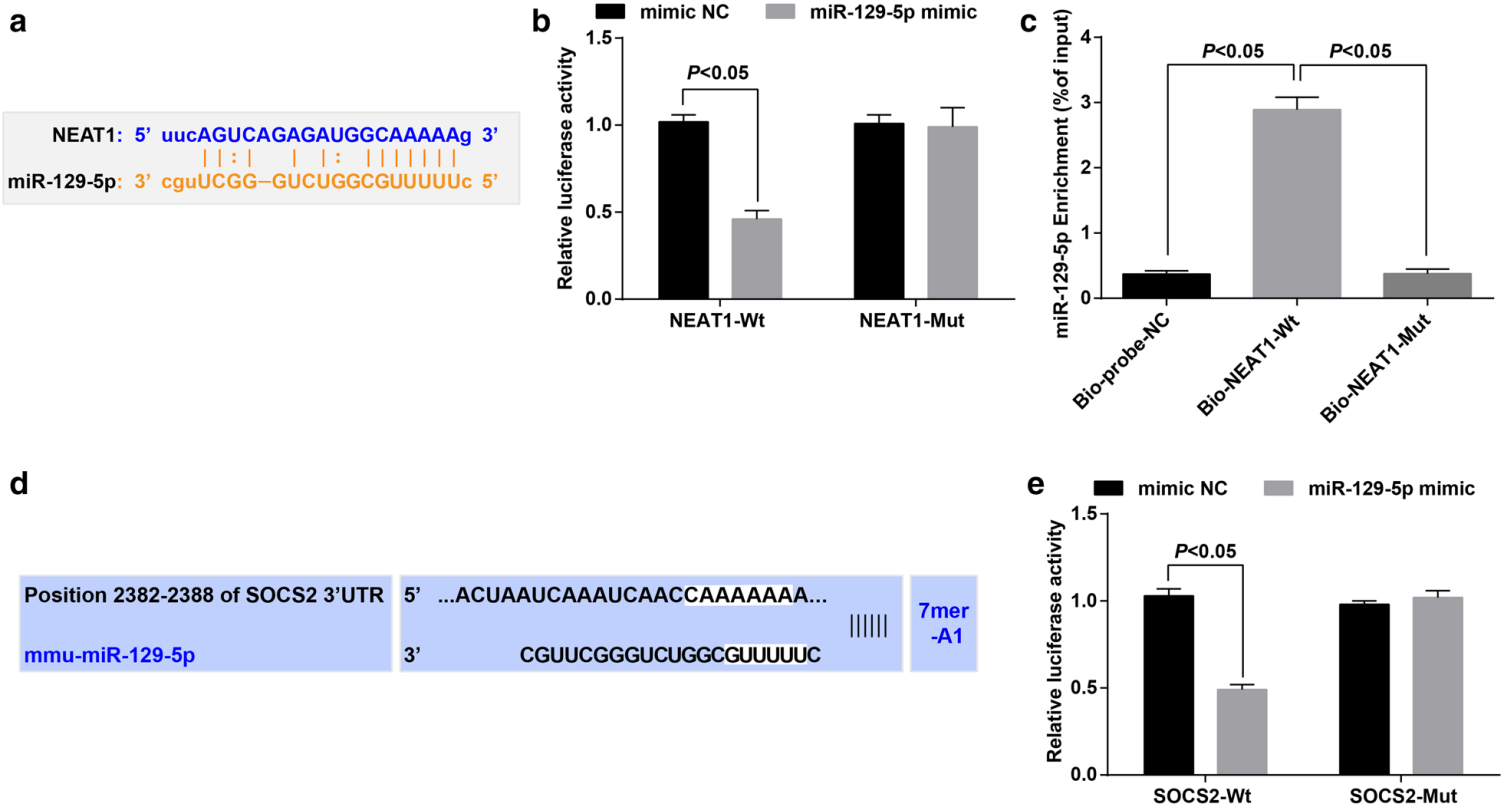
MiR-129-5p particularly binds with NEAT1, and SOCS2 is targeted by miR-129-5p. **a** binding sites of NEAT1 and miR-129-5p were predicted by https://starbase.sysu.edu.cn/; **b** binding relation between NEAT1 and miR-129-5p was confirmed by dual luciferase reporter gene assay; **c** effect of miR-129-5p on enrichment of NEAT1 was measured by RNA pull-down assay; **d** target relation between miR-129-5p and SOCS2 was predicted by https://www.targetscan.org/vert_72/; **e** target relation between miR-129-5p and SOCS2 was confirmed by dual luciferase reporter gene assay; the measurement data were expressed as mean ± standard deviation, the unpaired t-test was performed for comparisons between two groups, one-way ANOVA was used for comparisons among multiple groups, and Tukey’s post hoc test was used for pairwise comparisons after one-way ANOVA. The experiment was repeated for 3 times

According to the bioinformatic website (https://starbase.sysu.edu.cn/) (Fig. 6a), we have discovered that NEAT1 could bind with miR-129-5p. As confirmed by dual luciferase reporter gene assay (Fig. 6b), miR-129-5p overexpression markedly suppressed luciferase expression driven by pmirGLO-NEAT1-Wt but not that driven by pmirGLO-NEAT1-Mut, suggesting that miR-129-5p could bind with NEAT1. RNA pull-down assay further testified that bio-NEAT1-Wt could enrich more miR-129-5p in contrast to the bio-NEAT1-Mut and bio-probe-NC (Fig. 6c). We could conclude based on these results that NEAT1 served as a ceRNA to absorb miR-129-5p.

As predicted by the bioinformatic website (https://www.targetscan.org/vert_72/) (Fig. 6d), miR-129-5p could bind with SOCS2. The target relation between miR-129-5p and SOCS2 was confirmed by dual luciferase reporter gene assay (Fig. 6e), and the outcomes mirrored that the reporter with pmirGLO-SOCS2 3′-UTR-Wt exhibited weakened luciferase activity following co-transfection with miR-129-5p mimic. Nevertheless, the luciferase activity of the mutant reporter did not alter following miR-129-5p mimic transfection, indicating that SOCS2 was the direct target gene of miR-129-5p.

## Discussion

Belonging to the ALDs, ASH may develop into liver fibrosis, cirrhosis, liver failure, or even HCC, and it has been proved that the six-month mortality rate of severe ASH is 40% [3]. When it comes to the functional mechanisms of lncRNAs, the ceRNA hypothesis assumed that particular RNAs could impair the activity of miRNAs via sequestration, thus up-regulating their target genes [25]. Our research aimed to assess the role of the NEAT1/miR-129-5p/SOCS2 axis in ASH development, and we have discovered that the inhibited NEAT1 could repress liver fibrosis and development of ASH through the elevation of miR-129-5p and suppression of SOCS2.

Several results have been concluded in this study and one of them suggested that NEAT1 and SOCS2 were highly expressed while miR-129-5p was poorly expressed in serum of ASH patients and mice models. Similarly, Chen et al. have figured out that NEAT1 was considerably up-regulated in NAFLD models [9], and a same tendency of NEAT1 in hepatocytes under NAFLD condition has also been demonstrated [10]. Evidences have suggested that the miR-129-5p expression was restrained in HCC tissues or cells [13, 14]. As for the expression of SOCS2, a published literature has unveiled that the level of SOCS2 was elevated in rats that fed with Western style high-fat and high cholesterol diet, suggesting that SOCS2 was highly expressed in NAFLD rat models [19]. Moreover, we have found that NEAT1 could function as a ceRNA to sponge miR-129-5p, and the similar relation between NEAT1 and miR-129-5p has been elucidated in other researches as well [15, 16]. Bioinformatic method and dual luciferase reporter gene assay in our study indicated a target relation between miR-129-5p and SOCS2. However, this relationship remains uncovered.

Another result in our research indicated that knockdown of NEAT1 was able to improve the lipid metabolism by amplifying miR-125-9p and inhibiting SOCS2 in ethanol-stimulated AML-12 cells. In consistent with this outcome, Liu et al. have illuminated that the degradation of NEAT1 had the capacity to reduce the levels of diacylglycerol and free fatty acid in rat HCC models, thereby modulating the abnormal lipid metabolism in hepatocytes [26], and Zadjali et al. have pointed out that the inhibition of SOCS2 could protect against hepatic steatosis in mice that fed with high-fat-diet [20]. In addition, we have unraveled that the down-regulation of NEAT1 and up-regulation of miR-129-5p were able to restrict the inflammation reaction in ASH mice by reducing SOCS2. In accordance with this outcome, Wang et al*.* have figured out that the suppression of NEAT1 could attenuate inflammation response and lipid uptake in human macrophages [27], and it has been clarified by a recent document that the elevation of miR-129-5p relieved neuroinflammation after ischemia–reperfusion [28]. Furthermore, we have discovered that the reduced NEAT1 or amplified miR-129-5p could decelerate the apoptosis of hepatocytes in ASH mice by targeting SOCS2. In line with the result, a recent study has reported that the repression of NEAT1 inhibited the apoptosis of human kidney tubular cells in acute kidney injury [29], it has also been illustrated by Zhang et al. that miR-129-5p overexpression had the ability to inhibit apoptosis of cardiomyocytes in ischemic heart disease [30], and Xue et al*.* have discovered that the reduction of SOCS2 protected cardiomyocytes from apoptosis that induced by myocardial ischemia/reperfusion injury [31]. Additionally, we have evidenced that the suppressed NEAT1 and SOCS2, and the promoted miR-129-5p were able to attenuate the liver fibrosis in ASH mice. A similar finding has been noted by Yu et al. that the deletion of NEAT1 restrained liver fibrosis in vivo and in vitro [32], and it has been revealed by a recent research that the inhibition of miR-129-5p contributed to the collagen I synthesis in hepatic stellate cells, which is the pathogenesis of liver fibrosis [33].

## Conclusion

In conclusion, we have illustrated that the repression of NEAT1 and SOCS2, and upregulation of miR-129-5p could decelerate the development of ASH, which may provide novel evidence for the functional mechanisms of NEAT1/miR-129-5p/SOCS2 axis in ASH. We identified NEAT1 could play a regulatory role in liver function and provide novel insights into the modulation of hepatocyte viability in ASH. Moreover, this research also supplies the possibility of overcoming alcohol over-consumption-related diseases to some extent. Nevertheless, further studies are demanded to validate our results.

## Data Availability

Not applicable.
